# Timing of Peak Blood Glucose after Breakfast Meals of Different Glycemic Index in Women with Gestational Diabetes

**DOI:** 10.3390/nu5010001

**Published:** 2012-12-21

**Authors:** Jimmy Chun Yu Louie, Tania P. Markovic, Glynis P. Ross, Deborah Foote, Jennie C. Brand-Miller

**Affiliations:** 1 School of Health Sciences, Faculty of Health and Behavioral Sciences, The University of Wollongong, Wollongong, NSW 2522, Australia; E-Mail: jlouie@uow.edu.au; 2 School of Molecular Bioscience, Boden Institute of Obesity, Nutrition, Exercise and Eating Disorders, The University of Sydney, Sydney, NSW 2006, Australia; E-Mail: tania_markovic@ozemail.com.au; 3 Department of Endocrinology, Royal Prince Alfred Hospital, Camperdown, NSW 2050, Australia; E-Mail: gpross@bigpond.net.au; 4 Department of Nutrition and Dietetics, Royal Prince Alfred Hospital, Camperdown, NSW 2050, Australia; E-Mail: deborah.foote@sswahs.nsw.gov.au

**Keywords:** glycemic index, breakfast, gestational diabetes mellitus, pregnancy, peak blood glucose level

## Abstract

This study aims to determine the peak timing of postprandial blood glucose level (PBGL) of two breakfasts with different glycemic index (GI) in gestational diabetes mellitus (GDM). Ten women with diet-controlled GDM who were between 30 and 32 weeks of gestation were enrolled in the study. They consumed two carbohydrate controlled, macronutrient matched bread-based breakfasts with different GI (low *vs.* high) on two separate occasions in a random order after an overnight fast. PBGLs were assessed using a portable blood analyser. Subjects were asked to indicate their satiety rating at each blood sample collection. Overall the consumption of a high GI breakfast resulted in a greater rise in PBGL (mean ± SEM peak PBGL: low GI 6.7 ± 0.3 mmol/L *vs.* high GI 8.6 ± 0.3 mmol/L; *p* < 0.001) and an earlier peak PBGL time (16.9 ± 4.9 min earlier; *p* = 0.015), with high variability in PBGL time between subjects. There was no significant difference in subjective satiety throughout the test period. In conclusion, the low GI breakfast produced lower postprandial glycemia, and the peak PBGL occurred closer to the time recommended for PBGL monitoring (*i.e.*, 1 h postprandial) in GDM than a macronutrient matched high GI breakfast.

## 1. Introduction

Glucose that comes from the mother provides fuel for fetal growth [1]. High maternal blood glucose levels (BGL), even within the current recommended range, have been associated with higher infant body fat [2]. The peak postprandial BGL (PBGL) occurs later in pregnant women than in the non-pregnant state [3], *i.e.*, at 60 *vs.* 30 min, and is an important contributor to the risk of fetal overgrowth [4,5]. Treatment of gestational diabetes mellitus (GDM), where maternal glucose homeostasis is impaired, is therefore generally based on a combination of pre-meal BGL and PBGL 1 or 2 h after meals as monitored by self-blood glucose monitoring [6,7].

The glycemic index (GI) is a measurement of the glycemic quality of the carbohydrates in foods, where a low GI indicates that the carbohydrates are digested and absorbed slowly [8]. The limited evidence available suggests that GI of meals is of relevance in the setting of GDM [9]. A low GI diet was shown to reduce the requirement for insulin in the glycemic management of women with GDM [10], and was equally effective in improving pregnancy outcomes in GDM when compared to a conventional high fibre diet [11]. 

While the GI concept may be valid in diabetic pregnancy, scepticism of the efficacy of a low GI diet in GDM remains. This could partly be attributed to the lack of scientific evidence to demonstrate that low GI meals actually reduce, but not delay, the peak PBGL in GDM. A recent analysis of glycemic responses to over 1000 foods indicated that the timing of the peak was the same for high *vs.* low GI foods in non-diabetic individuals [12]. Whether this is true in pregnancy, especially those complicated by GDM, remains unclear because changes in gastric motility [13] and insulin sensitivity [14] during pregnancy may alter digestion and absorption. 

In this study we investigated whether two bread-based breakfasts with different GI produced different postprandial peaks and peak time points in a group of women with GDM. Our hypothesis was that a low GI bread-based breakfast would produce a lower but not delayed PBGL peak in GDM when compared to an energy and macronutrient matched high GI bread-based breakfast.

## 2. Experimental Section

### 2.1. Subjects

All women who attended the Royal Prince Alfred Hospital GDM antenatal clinic during June 2010 to November 2011 were approached for recruitment, and ten women aged 18–45 years, who had been diagnosed with GDM by a 75 g oral glucose tolerance test (OGTT) using the following criteria: fasting glucose 5.5 mmol/L or more and/or 1 h post-load glucose of 10.0 mmol/L or more and/or 2 h post-load glucose of 8.0 mmol/L or more; were between 30 and 32 weeks of gestation, with no known food allergy and/or special dietary requirement and not currently using insulin were enrolled in the study. Information about demographics and ethnicity was gathered. All women in this study received a standard GDM group education session with an experienced diabetes dietitian, which covered carbohydrate counting, importance of even distribution of carbohydrates throughout the day, and food sources of carbohydrate, with no specific emphasis of GI.

### 2.2. Anthropometry and Self Blood Glucose Monitoring (SBGM)

Subjects were weighed at the first study session in light clothing and with shoes off, and their height was obtained from their electronic medical record. Subjects were instructed to self-monitor their BGL using a glucometer (AccuChek Performa, Roche Diagnostic, Castle Hill, NSW, Australia). Fasting BGL on the 7 previous days was obtained from the electronic record of the glucometer. 

### 2.3. Test Meals

Subjects were required to fast for at least 8 h prior to the start of the test sessions. They consumed a carbohydrate controlled, low GI bread-based breakfast on one occasion, and an energy and macronutrient matched high GI bread-based breakfast on the other occasion one to two weeks apart. The order of test meals was randomized, and the allocation sequence was unpredictable and concealed from the recruiter. The subjects were asked to complete the meals within 15 min. The composition and nutritional content of the two test meals (Table 1) were analysed with FoodWorks Professional (version 2009, Xyris Software, Brisbane, Australia), using AUSNUT2007 as the source of nutrition composition [15].

**Table 1:** Composition and nutritional analysis of the test meals. ^a^ Burgen fruit and muesli bread; ^b^ Flora mono-sun margarine; ^c^ Devondale light dairy blend; ^d^ Benefibre fibre supplement; ^e^ Tip Top Sunblest wholemeal; ^f^ Lucozade orange flavour.

	Low GI	High GI
Foods included	70 g fruit bread ^a^	60 g wholemeal bread ^e^
	3 g margarine ^b^	134 g Fizzy glucose drink ^f^
	3 g light dairy blend ^c^	1 hardboiled egg
	200 mL skim milk	
	1.7 g fibre supplement ^d^	
*Nutritional Analyses*		
Energy (kcal)	328	328
Protein (% kcal)	18.9	18.3
Fat (% kcal)	22.1	24.7
Carbohydrates (% kcal)	54.5	52.1
Dietary Fibre (g)	4.2	3.9
Glycemic index	45	82
Glycemic load	21	36

### 2.4. Quantification of Fasting and Postprandial Blood Glucose Levels

Finger prick blood samples were collected before the start of the meal, and at 15, 30, 45, 60, 75, 90 and 120 min after the start of the meal. The blood samples were analysed immediately after collection using a portable blood analyser (HemoCue Glucose Analyzer 201, HemoCue Australia Pty Ltd., Wamberal, Australia). 

### 2.5. Subjective Satiety

Participants were asked to indicate their subjective satiety rating on a 15 cm visual analogue scale at each blood sample collection, with 0% representing “extremely hungry” and 100% representing “extremely full”.

### 2.6. Statistical Analyses

**Figure 1:**
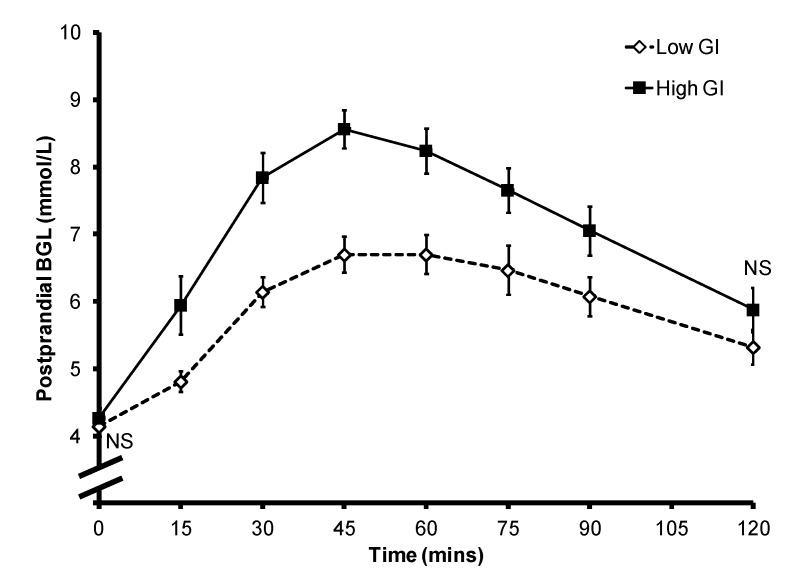
Mean ± SEM postprandial blood glucose level of the 10 subjects. NS: non-significant.

**Figure 2:**
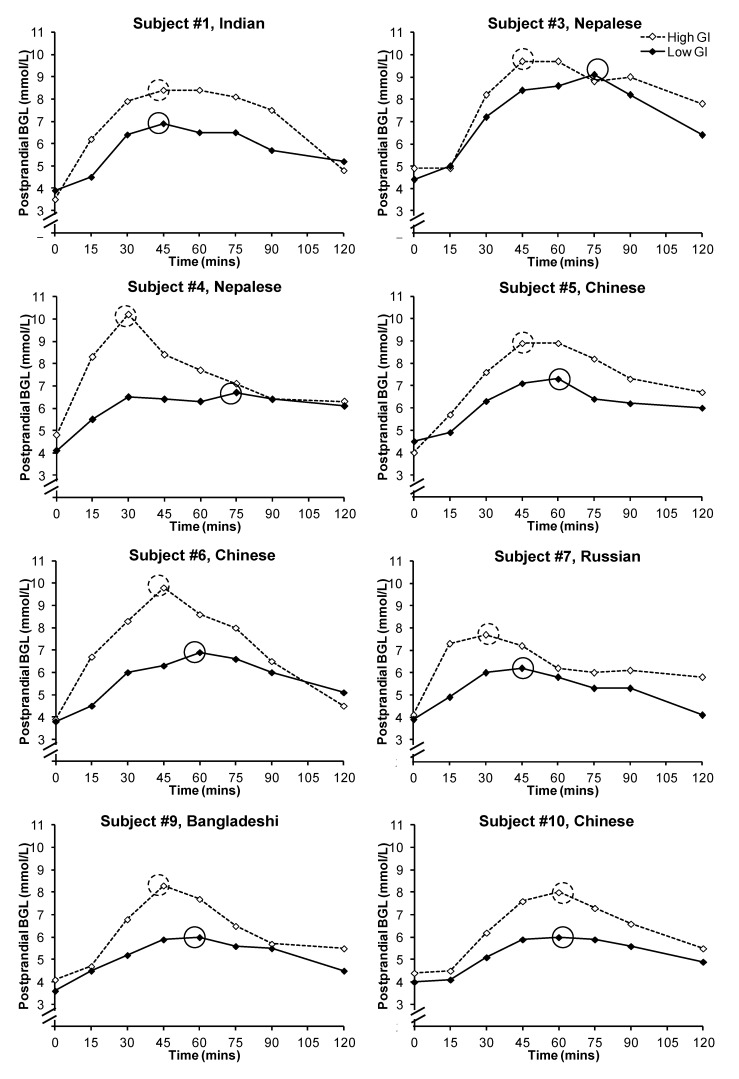
Postprandial glycemic responses of the subjects after the consumption of a low or high glycemic index breakfast. Peak blood glucose levels were circled. Subjects #2 and #8 withdrew after the first test session and hence their individual data were not presented.

All statistical analyses were performed in IBM SPSS version 19 (IBM Australia, St Leonards, Australia). Two women withdrew from the study after the first session, and their results were used only in the overall analysis (Figure 1). Mean ± SEM BGL for all subjects (Figure 1), as well as that of the individual subjects (Figure 2) were plotted against time to obtain postprandial blood glucose curves, and the incremental area under the glucose curve (iAUC_glucose_) was calculated by the trapezoidal rule. Paired sample *t*-test was used to compare differences in PBGL and subjective satiety between the breakfasts, and independent sample *t*-test was used to compare differences in postprandial iAUC_glucose_ between the breakfasts as two subjects only provided data for one breakfast. Their data were included in the analysis because the results did not differ significantly when they were excluded. The time point with the highest mean blood glucose level was deemed as the time of peak PBGL for the overall analysis (Figure 1), and the peak PBGL time for individual subjects were also identified (Figure 2).

### 2.7. Ethics Approval

This study was conducted according to the guidelines laid down in the Declaration of Helsinki and all procedures involving human subjects/patients were approved by the Human Research Ethics Committee of the Sydney South West Area Health Service (RPAH Zone). Written, informed consent was obtained from all subjects in this study.

## 3. Results

The study sample included five South Asians (Indian, Nepalese and Bangladeshi), four Chinese, and one Caucasian. The subjects attended the first and second session at a mean ± SEM of 33.5 ± 0.5 and 35.1 ± 0.6 weeks of gestation respectively. There was no difference in BMI (first visit: 24.9 ± 0.4 kg/m^2^* vs.* second visit: 26.1 ± 0.9 kg/m^2^; *p* = 0.212) and mean 7-day fasting BGL (first visit: 4.8 ± 0.1 mmol/L *vs.* second visit: 4.8 ± 0.1 mmol/L; *p* = 0.461) on the test days. No subject started insulin therapy during the study period. 

**Figure 3:**
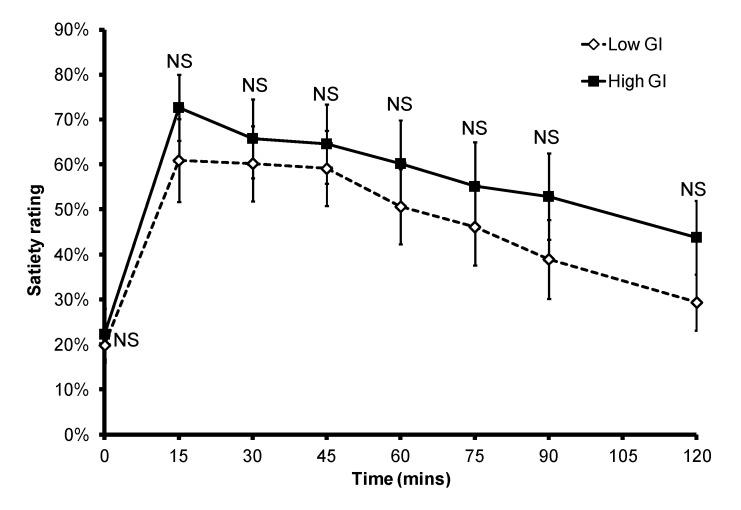
Subjective satiety after a low or high GI breakfast. NS: non-significant.

Overall the consumption of a high GI bread-based breakfast resulted in higher postprandial glycemia (Figure 1). The mean ± SEM iAUC_glucose_ after a low GI breakfast was significantly lower than that after the consumption of a high GI breakfast (212.7 ± 22.9 *vs.* 340.8 ± 23.4; *p* = 0.001). The mean ± SEM peak BGL was 6.7 ± 0.3 mmol/L for the low GI breakfast and 8.6 ± 0.3 mmol/L for the high GI breakfast (*p* < 0.001). However, there was large inter-subject variability in the timing of the peak between the two test meals (Figure 2): the peak occurred between 45 and 75 min for the low GI breakfast (mean ± SEM: 60.0 ± 4.0 min), and between 30 and 60 min for the high GI breakfast (mean ± SEM: 43.1 ± 3.4 min; *p* = 0.015). In the eight subjects who provided data for both breakfasts, six had a delayed peak PBGL time after consuming the low GI breakfast when compared to that of the high GI breakfast. There was no significant difference in subjective satiety throughout the 2-h test period (Figure 3).

## 4. Discussion

This study is the first attempt to examine the timing of the peak PBGL in women with GDM after consumption of breakfasts of different GI values. We expected to see differences in the peak concentration but similar timing of the peak. However we found that the low GI breakfast produced a peak at closer to 60 min after the start of the meal compared with ~45 min after the high GI breakfast.

It has been previously shown that a low GI diet produces comparable outcomes as a conventional high fibre diet in pregnancy complicated with GDM [11]. The limited evidence on the efficacy of a low GI diet for the management of GDM has suggested that a low GI diet may improve postprandial glycemia [10,16], therefore reducing excessive transfer of maternal blood glucose to the fetus. In GDM, breakfast was shown to have the greatest variability in postprandial glycemic response [17]. The results from our study suggested that a low GI breakfast may be of benefit in the management of post-breakfast glycemia. 

Since peak PBGL has been shown to be more strongly associated with outcomes of GDM pregnancy [18] than 2-h PBGL, it is important to accurately time the postprandial SBGM testing to capture the peak BGL. In healthy, non-pregnant subjects low and high GI meals reach peak concentrations at the same time, *i.e.*, at 30 min [12]. However during pregnancy, especially those complicated with GDM, changes in gastric motility [13] and insulin sensitivity [14] can be expected to alter the rate of carbohydrate digestion and absorption, and hence shape of the postprandial glucose curve. Indeed, Wolever *et al.* [19] showed that the peak PBGL after a standard test meal occurred later and later as glucose tolerance worsened in subjects with diabetes.

A previous study had found that the peak PBGL in GDM occurs at about 60 min post meal [7]. Therefore SBGM that tests the 2 h PBGL may miss the glucose peak, and the clinical decision to commence insulin therapy was usually based on a cut-off of 1-h PBGL [6]. We found that while a low GI breakfast delayed the peak PBGL in GDM, the peak occurs closer to 60 min after the start of the meal than a high GI breakfast, which produced a peak at ~45 min post meal. Therefore our results suggested the findings of the study by Moses *et al. *[10], which demonstrated that a low GI diet reduces the need for insulin in GDM for BGL management, was indeed due to the fact that low GI meals produce a lower peak PBGL at 60 min post meal. Our results also suggest that the 1-h postprandial SBGM reading is likely to underestimate the actual postprandial glycemic response to a high GI meal. Therefore our finding raises the question whether the GI of the patient’s diet should be considered in the interpretation of SBGM results. 

Our study has limitations, including a small sample size and a high proportion of Asian subjects, which compromises the generalizability of the findings. In addition, the non-continuous nature of blood sample collection did not allow accurate determination of the actual peak time. However, more frequent fingerprick blood sampling would be impractical. Although continuous glucose monitoring might be helpful in this context, it measures changes in the interstitial fluid rather than capillary blood, and by nature is subject to a delay [20,21,22]. 

## 5. Conclusions

The low GI breakfast produces lower postprandial glycemia than a macronutrient matched high GI breakfast. The timing of the peak BGL varies within and between breakfasts of different GI. The peak PBGL after a high GI breakfast occurs at ~45 min, 15 min earlier than that of a low GI breakfast. The peak PBGL of a low GI breakfast occurs closer to the time recommended for PBGL monitoring in GDM. Since many women consume high GI meals throughout pregnancy, there are implications for clinical practice.
